# The Clinical Guide

**Published:** 1891-12

**Authors:** 


The Clinical Guide; or, Pocket Repertory For the Treatment of Acute and Chronic Diseases. By G. H. G. Jahr, Translated by Charles J. Hempel, M. D., Second American, Revised and Enlarged from the Third German Edition, enriched by the addition of the New Remedies, by Samuel Lilienthal, M. D. Philadelphia: Hahnemann Publishing House, 1891. Price, $3.00.

No homœopathic physician can see this book, but buy it. It is a timely book and contains a mine of wealth. Like the Professor of Lutheran theology, who was in the habit of saying to his students, when telling them of the priceless value of Luther's books: "Gentlemen, if you should have to do without an overcoat, buy Luther's works." So we say to every member of our school: buy the new edition of Jahr's *Clinical Guide*, though on that account you were to miss your favorite cigar for a few weeks.

The paper, printing, and binding are excellent. W. S.

